# Clinician and cancer patient views on patient participation in treatment decision-making: a quantitative and qualitative exploration

**DOI:** 10.1038/sj.bjc.6604611

**Published:** 2008-08-19

**Authors:** A H Pieterse, M C M Baas-Thijssen, C A M Marijnen, A M Stiggelbout

**Affiliations:** 1 Department of Medical Decision Making, University Medical Center Leiden, Leiden, The Netherlands; 2 Department of Radiotherapy, Netherlands Cancer Institute, Amsterdam, The Netherlands; 3 Department of Clinical Oncology, University Medical Center Leiden, Leiden, The Netherlands

**Keywords:** patient values, treatment guidelines, treatment preferences, shared decision-making, adjuvant treatment

## Abstract

Patient participation in treatment decision-making is being increasingly advocated, although cancer treatments are often guideline-driven. Trade-offs between benefits and side effects underlying guidelines are made by clinicians. Evidence suggests that clinicians are inaccurate at predicting patient values. The aim was to assess what role oncologists and cancer patients prefer in deciding about treatment, and how they view patient participation in treatment decision-making. Seventy disease-free cancer patients and 60 oncologists (surgical, radiation, and medical) were interviewed about their role preferences using the Control Preferences Scale (CPS) and about their views on patient participation using closed- and open-ended questions. Almost all participants preferred treatment decisions to be the outcome of a shared process. Clinicians viewed participation more often as reaching an agreement, whereas 23% of patients defined participation exclusively as being informed. Of the participants, ⩾81% thought not all patients are able to participate and ⩾74% thought clinicians are not always able to weigh the pros and cons of treatment for patients, especially not quality as compared with length of life. Clinicians seemed reluctant to share probability information on the likely impact of adjuvant treatment. Clinicians should acknowledge the legitimacy of patients' values in treatment decisions. Guidelines should recommend elicitation of patient values at specific decision points.

Both clinicians and researchers have commented on the need to involve patients in treatment decision making, especially when a patient presents with a serious illness, different treatment options exist, the gains of treatment should be weighed against possible adverse effects, or outcomes are uncertain (Guadagnoli and Ward, 1998; Charles *et al*, 1999b; Robinson and Thomson, 2001). Involving patients in treatment-related decision making is in line with the increasingly acknowledged patients' right to autonomy and self-determination. The need to involve patients is supported by evidence that physicians do not have the ability to adequately judge patients' values for outcomes of care (Cotler *et al*, 2001; Montgomery and Fahey, 2001; Brothers *et al*, 2004; Stalmeier *et al*, 2007).

Treatment in oncology is often guideline-based, at least in the Netherlands. Clinicians may access up-to-date information on nationwide guidelines in oncological and palliative care through http://www.oncoline.nl, a publication of the Dutch Association of Comprehensive cancer centres (http://www.ikcnet.nl). In the guideline development process, trade-offs between expected benefits and side effects are almost exclusively made by clinicians, not by patients. Also, these trade-offs are usually not made explicit to patients. If patient values are to be incorporated in treatment decision making, which seems especially relevant in decisions on adjuvant treatment, this requires patients to participate in the consultation and voice their values. There is considerable uncertainty about what patients and clinicians understand by patient participation (Guadagnoli and Ward, 1998). This study was set up to assess what role oncologists and cancer patients prefer in deciding about cancer treatment, and how they view patient participation in treatment-related decision making. Earlier studies have assessed decisional role preferences, but these are of limited interest to situations where treatment choices are mostly guideline-driven.

## Materials and methods

### Study population

Participants were disease-free rectal cancer patients who had participated in a study assessing rectal cancer treatment preferences (Pieterse *et al*, 2007). For that study, a stratified random sample was selected from patients who had participated in a multicenter trial assessing the benefit of adding preoperative radiotherapy (PRT) to total mesorectal excision surgery between January 1996 and December 1999 (Kapiteijn *et al*, 2001). Stratification was carried out to include equal numbers of patients from both treatment groups as well as those who were reported to suffer from side effects at follow-up in both groups. A total of 94 eligible patients were approached. Of these, four patients could not be reached and nine had other types of cancer or recurrent disease and were excluded. Of the remaining 81 patients, 70 (86%) agreed to participate. Reasons for refusal were the psychological burden (*N*=8), physical burden (*N*=1), time investment (*N*=1), or unknown (*N*=1).

For the treatment preference study, we aimed further to include 60 oncologists specialised in gastroenterology. We predominantly aimed for surgical and radiation oncologists, as these specialties are most involved in primary rectal cancer treatment. Seventy eligible oncologists were randomly selected from surgeons, radiotherapists, and medical oncologists involved in the Simply Capecitabine in Rectal cancer after Irradiation Plus TME (SCRIPT) trial and were approached. Three clinicians could not be reached. Sixty (86%) oncologists agreed to participate (25 surgical, 25 radiation, and 10 medical). Reasons for refusal were time constraints (*N*=4), considering participation not meaningful (*N*=1 medical oncologist), being retired (*N*=1), or unknown (*N*=1).

### Procedure

Eligible participants were informed about the study by letter and then asked by phone whether they agreed to participate. One of two trained interviewers (AHP and MCMB-T) conducted individual face-to-face interviews following a strict protocol. Interviews were held at home (patients) or at the institution (oncologists). All patients gave written informed consent at the start of the interview. Interviews with clinicians were audiotaped with their permission. Prior to the interview, sociodemographic, disease- (patients) and work- (oncologists) related data were collected using a self-administered paper-and-pencil questionnaire. The medical ethical board of the Leiden University Medical Centre approved the study. Participants were included between February and August 2006.

### Interviews

We started the interview by assessing role preferences in treatment decision-making using the Control Preferences Scale (CPS) (Degner and Sloan, 1992). The CPS was devised to assess the degree of control an individual wants to assume when decisions are being made about a specific medical treatment (Degner *et al*, 1997b). It has been widely used in previous studies of cancer patients (Rothenbacher *et al*, 1997; Wallberg *et al*, 2000; Mallinger *et al*, 2006). We started out with this measure to help participants think about participation in decision making. Patients especially may never have thought about such issues. We asked participants to focus on the hypothetical decision whether or not to undergo PRT, had they presently been diagnosed with rectal cancer (patients), or were they seeing a patient diagnosed with rectal cancer (oncologists). The decision was not familiar to patients, as they had been randomised to receive either PRT or not at the time they were actually treated. Notably, we had not informed patients about benefits and side effects of PRT.

**Figure 1:**
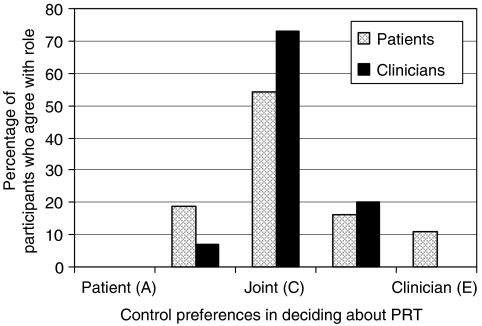
Patients' (*N*=70) and clinicians' (*N*=60) control preferences in deciding about preoperative radiotherapy (PRT). Note: Phrasing of control preferences roles in *patient* interviews: (A) I prefer to make the decision about my treatment; (B) I prefer to make the decision about my treatment, after seriously considering my doctors' opinion; (C) I prefer that my doctor and I make the decision about my treatment jointly; (D) I prefer that my doctor makes the decision about my treatment, after seriously considering my opinion; (E) I prefer to leave the decision about my treatment to my doctor. Phrasing of control preferences roles in *clinician* interviews: (A) I prefer to leave the decision about treatment to my patient; (B) I prefer that my patient makes the decision about treatment, after seriously considering my opinion; (C) I prefer that my patient and I make the decision about treatment jointly; (D) I prefer to make the decision about treatment, after seriously considering my patient's opinion; (E) I prefer to make the decision about treatment.

The CPS consists of five cards, each of which portrays a different role in treatment decision making using a statement. The roles ranged from (A) the patient deciding on PRT, through (C) the individual making the decision jointly with the physician (or patient), to (E) the physician making the decision about PRT (Figure 1). Participants were presented with a series of paired comparisons of roles until the preferred role was established, following the same procedure as Davey *et al* (2004). The statements thus indicate a preference for an active (A and B), collaborative (C), or passive (D and E) patient role in treatment decision making.

Participants were then asked a set of closed questions relating to the desirability of patient participation and to the weighing of pros and cons of treatments (Table 1). Clinicians were asked whether they would offer the option of an adjuvant treatment that cures an additional 1–5% patients, but with a clinically relevant risk of side effects in patients treated with that adjuvant treatment (the absolute numbers and the nature of the treatment and side effects were not further specified). The example was chosen as a general case for current adjuvant treatments in cancer care. Clinicians were further questioned about their preference regarding the role of patients in the formulation of treatment guidelines. Participants were asked to explain their answer following each question. The interviewers noted participants' answers on paper.

### Coding

A random selection of five taped clinician interviews was used to compare the recorded explanations with the paper notes taken during the interview. This showed that main themes had not been missed on paper.

Answers to the open-ended questions were categorised. Each of the interviewers read a different random selection of five patient interviews and separately developed an initial list of codes intended to reflect the various views in participants' responses. The interviewers compared their list of codes and decided on a definitive set of codes and subheadings. In coding oncologists' answers, additional codes were decided on as needed. Both interviewers then coded all participants' explanations, reviewed their coding, and resolved disparities. Participants' answers could be categorised into one or more categories (see Appendix A), depending on the number of reasons they nominated. Answers were not categorised if they reiterated a previous ‘yes’ or ‘no’ answer to the closed-ended question, if it was open to interpretation, or if it did not refer to the question but went beyond.

### Analysis

Descriptive statistics (frequencies and percentages) were used to describe sociodemographic, disease- (patients) and work- (oncologists) related data, role preferences, and the answers to the closed- and open-ended questions. Using Fisher's exact or *χ*^2^ tests, as appropriate, proportions of patients and clinicians were compared on their answers to the closed-ended questions and on how often their explanation fell into one of the coding categories. Bivariate associations between participants' characteristics and role preferences (active, collaborative, and passive patient role) and responses to question 1 (Table 1) were assessed using *t*-tests, ANOVA, Kendall's *τ*-correlation, *χ*^2^ tests, and Fisher's exact tests. Significance testing was done two-sided at *α*=0.05.

## Results

### Participants

Forty-eight male and 22 female patients participated. They were aged 64 years on average (s.d.=9.4; range: 41–84) at the time of the interview and had been treated for rectal cancer 6–10 (M=8; s.d.=1.0) years ago. Among those responding to demographic questions, 31 (45%) patients had completed 9 years or less of education, 23 (33%) 10–12 years, and 15 (22%) 13 years or more. All patients had undergone surgery and 38 (54%) had also been treated with PRT.

All surgeons as well as 14 out of 25 radiation and 9 out of 10 medical oncologists were male. The clinicians were between 35 and 62 years old (M=48, s.d.=7.3). Time since specialisation was 13 years on average (s.d.=8.1, range, 1–31) and was not significantly different according to specialty.

### Control preferences scale

Figure 1 depicts participants' preferences regarding their role in the decision about PRT in the treatment of rectal cancer. Except for eight (11%) patients who preferred to leave the decision to their clinician, all participants would prefer both the patient and the clinician to share in that decision.

There was a trend (*P*=0.05) for patients' and clinicians' preferences over the active, collaborative, and passive patient roles to differ. The clinicians preferred in majority the collaborative role (73%), whereas patients' role preferences were more equally spread out over the three levels of involvement.

There was a significant association (*P*=0.03) between a lower educational level in patients and their preference to relinquish decisional control to their clinician. Also, preferences of male compared with female patients were significantly (*P*=0.04) different, with preferences of male patients being more equally distributed over the decisional roles. Clinicians' specialty was significantly (*P*=0.01) related to their role preferences. Medical, radiation, and surgical oncologists preferred the clinician to decide mainly in increasing proportions (10, 20, and 24%, respectively). No significant associations were found between decisional role preferences and participants' age, patients' past treatment with PRT (yes/no), or clinicians' time since specialisation.

### Desirability of patient participation in treatment-related decision making

Table 1 shows that overall almost all patients (*N*=66, 96%) and clinicians (*N*=57, 95%) thought that cancer patients should be involved in decision making regarding treatment (question 1). Responses did not significantly differ according to patients' gender, educational attainment, or past treatment, nor to clinicians' specialty.

The patients and clinicians did not significantly differ in the frequency with which they explained their answer by referring to patient autonomy (patients: 44%, clinicians: 46%), the need to inform patients (patients: 39%, clinicians: 35%), and to openness in patient–clinician communication (patients: 14%, clinician: 5%). A significantly larger proportion of clinicians than patients (23 *vs* 5%; *P*<0.01) referred to the outcome of the decision process, in that patients and clinicians should reach an agreement about treatment and/or that a participating patient is more motivated and therefore endures the treatment better. There was a trend for clinicians to refer more often to their responsibility and expertise (25 *vs* 12%; *P*=0.10). Eight (14%) clinicians compared with none of the patients referred to the necessity to comply with the informed consent procedure, and for six of the eight this was their sole explanation. Of the 66 patients, 15 (23%) motivated their response only as the need for patients to be informed, compared with none of the clinicians.

Most participants (patients: *N*=51, 85%; clinicians: *N*=46, 81%) thought that not all patients are able to participate in such decision making (Table 1, question 2). There was a trend (*P*=0.07) for patients' and clinicians' responses to differ if we took the patient (male, 71 years old, with 13 years or more education, and irradiated prior to surgery) into account who did not have an opinion on the issue. If we discard this participant, patients' and clinicians' responses did not significantly differ.

There was a trend (*P*=0.10) for patients (22%) to refer more often to patients' psychological inability (not further specified), when compared with clinicians (9%). Patients (43%) and clinicians (33%) referred in similar proportions to emotional incapacities, including patients' anxiety or lack of confidence. A significantly larger proportion of clinicians compared with patients (59 *vs* 22%; *P*<0.001) referred to cognitive deficiencies, in that patients may not grasp the information provided and especially have difficulties with understanding the risks involved. Clinicians tended also to refer more often to co-morbidity, including dementia and psychiatric disorders, when compared with patients (28 *vs* 12%; *P*=0.07). Other explanations related to sociodemographic factors, including age, social support, and religion (patients: 12%, clinicians: 20%) and the novelty and complexity of the decision situation for patients (patients: 6%, clinicians: 4%).

A large majority (patients: *N*=55, 79%; clinicians: *N*=41, 69%) of participants indicated that clinicians should try to involve patients in decisions about their treatment, even if the patient is reluctant to be involved (Table 1, question 3). Patients' and clinicians' responses significantly differed (*P*=0.04) if we take into account the two male respondents (70 and 75 years old, 9 years or less education, and irradiated preoperatively) who did not have an opinion on the issue. If we discard their response, patients and clinicians did not significantly differ in their opinion on the involvement in treatment decision making of reluctant patients.

Participants justified their conviction by referring to patient responsibility (patients: 18%, clinicians: 7%) and the temporary nature of patient's reluctance (patients: 7%, clinicians: 2%). Others (patients: 13%, clinicians: 15%) viewed it as part of the clinician's task. Moreover, participants (patients: 46%, clinicians: 56%) often referred to the manner in which physicians may proceed, such as by providing information, reassuring the patient, or involving significant others of the patient. Several participants (patients: 16%, clinicians: 17%) stated that the clinician should try to involve a reluctant patient only within reason. In contrast, four patients compared with none of the clinicians considered that a clinician should try to involve a patient to the utmost. Participants' reasons for not trying to involve a reluctant patient referred to respect for the patient's wish not to participate. Participants indicated, moreover, that in such circumstances a clinician should adopt the role of providing information (*N*=5) or involving significant others of the patient (*N*=1).

### Weighing benefits and side effects of treatments

A large majority of patients (*N*=52, 74%) and clinicians (*N*=49, 82%) thought that clinicians are not always able to weigh the pros and cons of treatment for their patients (Table 1, question 4).

Participants referred to individual differences in patients' experience and the acceptance of treatments (patients: 39%, clinicians: 35%), the lack of skills and/or the subjective stance of the clinician (patients: 21%, clinicians: 29%), and insufficiencies in the dialogue between patients and clinicians (patients: 27%, clinicians: 29%). Participants thinking that a clinician always has this ability (patients: *N*=16, 23%; clinicians: *N*=11, 18%), in contrast, referred mostly to clinicians' skills and responsibility (patients: 81%, clinicians: 55%). Others referred to the clinician knowing patients sufficiently (patients: 13%, clinicians: 27%). One patient (7%) explained it by the patient's trust in the clinician.

Overall, even higher proportions of participants (patients: *N*=61, 88%; clinicians: *N*=52, 87%) did not think that clinicians can always weigh the value of quality compared to length of life accurately for individual patients, even though this may be viewed as a specific case of weighing the pros and cons of treatment mentioned above (Table 1, question 5).

Participants related clinicians' lack of insight in patient values often to individual patient differences. Patients did so significantly more often than clinicians (patients: 66 *vs* clinicians: 42%; *P*=0.02). A significantly larger proportion of clinicians compared to patients underlined the importance of dialogue, referring to the need for patients to bring their values forward in the interaction with the clinician (patients: 10 *vs* clinicians: 35%; *P*<0.01). Other participants (patients: 7%, clinician: 10%) referred to limitations resulting from physicians' emotions, experience, and subjective stance.

When asked explicitly about an adjuvant treatment that cures an additional 1–5% patients at the expense of a clinically relevant risk of side effects, a significant minority (17%) of clinicians would not even propose the treatment to their patient. None of the remaining 83% clinicians would prescribe that treatment without discussing it first with their patient. Of them, half (54%) would tell patients the absolute probabilities of benefits and side effects and other half (46%) would not. Moreover, a majority (63%) of clinicians indicated that in their opinion, patients should be involved in the formulation of treatment guidelines. A significant minority (37%) was opposed to patient involvement at that stage.

## Discussion

The results from this study suggest that both in case of a specific adjuvant treatment decision situation and more generally, a large majority of treated cancer patients and clinicians prefer the decision to be the outcome of a shared decision-making process. Our results support previous research in newly diagnosed cancer patients (Degner *et al*, 1997a), in palliative cancer care patients (Rothenbacher *et al*, 1997), and in a healthy population (O'Donnell and Hunskaar, 2007) that a higher educational attainment is associated with a preference for a more active role in decision making. Our finding that gender may affect decisional role preferences adds to the evidence that is, as of yet, inconclusive (reviewed by Hubbard *et al*, 2008). Further assessments are needed before firm conclusions can be drawn. Our results on surgical oncologists preferring to a greater extent that the clinician mainly decides on treatment compared with radiation and medical oncologists may seem to diverge from Charles *et al*'s (2003) results. They found breast cancer surgeons to agree more often than radiation and medical oncologists to the statement that patients and physicians agreeing together to the treatment to be given, to be important to a shared decision-making process. However, surgeons' understanding of a shared process may differ from their preferences regarding actual decision-making in the clinic. Other determinants of decisional role preferences could not be traced.

Up to one-fifth of the patients and one-third of the clinicians felt that reluctant patients need not be involved in the decision making, referring to respect for the patient's attitude. Bearing in mind that clinicians are not accurate in judging patient values for the outcome of care (Cotler *et al*, 2001; Montgomery and Fahey, 2001; Brothers *et al*, 2004; Stalmeier *et al*, 2007), it seems in these cases critical to ascertain that the patient persists in preferring non-involvement.

Importantly, these results do not as such shed light on whether patients and clinicians actually agree on how patient involvement should take place. The participants provided a variety of explanations for their opinions on participation. Clinicians more often than patients defined patient participation in terms of clinician–patient communication, namely reaching an agreement about treatment. Some clinicians compared to none of the patients explained the need for patient participation in terms of the need to comply with the informed consent procedure. For a significant minority (10%) of clinicians, this was their only motivation. It is unclear how these clinicians view the legal definition of patient participation and whether it goes beyond informing patients and asking them for consent to treatment plans that clinicians have proposed.

There is no agreement on the definition of shared decision making in the literature (Makoul and Clayman, 2006; Moumjid *et al*, 2007). However, essential elements incorporated in all prominent cited models of shared decision making include not only the presentation of options but also the discussion of patient values (Makoul and Clayman, 2006). Accordingly, the focus of common definitions of shared decision making is both on information exchange between physician and patient and the involvement of both parties in the decision made (Moumjid *et al*, 2007). As a result, patient participation presupposes that both clinicians and patients have responsibilities, which stands in sharp contrast to patients only being informed. Strikingly, a significant minority of patients explained participation in decision making exclusively in terms of being informed. Our finding is in accord with evidence from a qualitative study including 41 patients diagnosed with colorectal cancer. Patients in that study reported to perceive that there was a ‘right’ decision to be made and that doctors would make the right decision for them (Beaver *et al*, 2005). Several other studies have also shown that patients more often prefer to receive information than to actually participate in decision making (Blanchard *et al*, 1988; Ende *et al*, 1989; Nease and Brooks, 1995; Benbassat *et al*, 1998). Clearly, not all patients want to be involved (Davison *et al*, 1995; Beaver *et al*, 1999; de Haes, 2006). Schwartz (2004) has even questioned the superiority of patient autonomy. Making choices may be a burden rather than a good option. By choosing one option, the patient dismisses the advantages of the options that are not selected, and this implies loss. Similarly, anticipated and post-decision regret may be at stake (de Haes, 2006). None of the clinicians, in contrast, appeared to agree that informing a patient is sufficient to consider the patient involved in the process. This finding is in line with results of Charles *et al* (2003), showing that only few physicians would define shared decision making in terms of information exchange alone.

Where clinicians and patients wish to share in the decision-making process, patients should voice their values during their interaction with their clinician. However, patients may feel intimidated by their doctor because of the power differential (Bryant *et al*, 2006), and therefore refrain from participating (Guadagnoli and Ward, 1998; Roter *et al*, 2007). Physician use of supportive communication was found to be essential to facilitate patient involvement (Street *et al*, 2005) and physicians' explicit encouragement of patient participation may foster patient participation in medical decision making (Fraenkel and McGraw, 2007).

More than 80% of patients and clinicians doubted as to all patients' ability to participate in treatment decision making, even though almost all participants considered it necessary. Clearly, participants' doubts as to patients' capacities could point to the exceptional cases of emotional disturbance or cognitive deficiencies. But the limitations that participants stated could apply to any ordinary patient, not the isolated case. Patients and clinicians often nominated emotional barriers. Indeed, distress may hamper patients' capacity to process information (McHugh *et al*, 1995; Erblich *et al*, 2003). Clinicians more often than patients further viewed cognitive limitations in patients as a barrier. Limited educational attainment and literacy skills have been shown to relate to difficulties in understanding and recalling complex medical information (Williams *et al*, 2002). As already suggested by some of the patients and clinicians in this study, clinicians may find ways to involve the more common patients, such as by addressing their fears, simplifying the information, or repeating it to facilitate its processing. Limited skills in patients to understand medical information should be an important motivation for clinicians to explain the information in even more simple language, take time to repeat the relevant details and to check understanding, and supply reference materials. Importantly, where inability is related to feelings of intimidation or lack of encouragement, clinicians could help patients to overcome patients' hesitations.

Interestingly, none of the participants in our study suggested time constraints as a barrier to patient involvement. In Fraenkel and McGraw's (2007) study, time during medical encounters was nominated as an essential element to enable patients to become informed and to process information. Results in patients with heterogeneous cancers suggest though that clinical encounters need not be lengthened if the clinician proactively addresses patient questions (Brown *et al*, 2001).

Patients and clinicians seemed to agree that clinicians need patient input to assess accurately how the individual patient weighs pros and cons of treatment alternatives. Up to one-fifth of patients and almost one-third of clinicians made reference to clinicians' subjective stance. They recognised that the expertise of clinicians does not exist in a vacuum but is embedded in their interpretation of the situation and their perception of the patient. Interestingly, if pros and cons are specified as quality *vs* length of life, participants agreed even more strongly to clinicians' limitations in this regard. In their explanations, patients emphasised individual patient differences. Clinicians underlined patients' role in terms of the need for clinicians to receive patient input in order to assess accurately how an individual patient weighs risks and benefits of treatments. In practise, this standpoint implies that clinicians are willing to inform patients about risks and benefits of treatments, and maybe to a larger extent than they routinely do. In particular, this requires the use of precise vocabulary so as to facilitate patient recall of the discussion of relevant treatment options (Keating *et al*, 2003) and guarantee the necessary specificity to help patients estimate the impact of the treatment on their lives (Davidson *et al*, 2007). This perspective also requires clinicians to help patients to bring their values forward. Evidently, clinicians should then be prepared to acknowledge the legitimacy of patients' values in treatment decisions. Also, patients should then accept to share the responsibility for the treatment decision (Charles *et al*, 1999a).

A significant minority of clinicians indicated that they would decide against offering an existing treatment if the small probability of extra benefit would go hand in hand with a large probability of side effects. This is what may be termed a physician's silent decision (Whitney and McCullough, 2007) and may be justified by considerations of (lack of) clinical utility. Yet, clinicians' opinion about the clinical utility in this case varied. Also, almost half of the clinicians who would offer the treatment, would not state the absolute probabilities. These results are in line with those from Ravdin *et al* (1998). Their study showed that women with breast cancer often are not given quantitative estimates of the magnitude of probable benefit and toxicity of adjuvant therapy. Not stating the probably impact of adjuvant therapy or using probability words instead of numbers may result in patients understanding poorly the trade-offs in benefits and side effects. Indeed, the breast cancer patients in Ravdin *et al*'s study overestimated their risk of early relapse and the effectiveness of their adjuvant therapy, even though they were younger and better educated than the average woman with breast cancer. For patients to be active, informed participants in the treatment-related decision-making process, probability information on benefits and risks of treatment alternatives is of critical value. It is questionable to what extent a patient can actually think about the pros and cons of treatment if the clinician is not willing to share relevant evidence.

Our results suggest that at least some clinicians support the involvement of patients in the formulation of guidelines. Alternatively, guidelines may explicitly prescribe the need to elicit patient views at specific decision points. Dutch guidelines on cancer treatment (www.oncoline.nl) including for example the recently updated guideline for rectal cancer and the one for breast cancer, include the recommendation that clinicians should extensively inform their patients about benefits and side effects of treatment alternatives. This recommendation underlines the need for clear information but does not go as far as stating that patient values for outcomes of care should explicitly be discussed when a treatment decision needs to be made, nor that treatment advice could vary, according to patient values. Yet, in order to obtain the best achievable care for individual patients, treatment choice should vary according to clinical circumstances and to patient values (Hlatky, 1995). In the process of involving patients further than only informing them, patients may increasingly prefer to be treated in other ways than standard treatment. Guidelines may be explicit on this point and include the remark that given particular patient values, deviating from consensus treatment may be acceptable.

In conclusion, our results run counter to guideline-based treatment of patients, in cases where clinicians' treatment advice is not based upon individual patient values. A majority of clinicians and patients in this study were in favour of clinicians and patients reaching an agreement on treatment. They held the conviction that clinicians cannot accurately predict how pros and cons of treatment weigh for their patients. Clinicians should inform patients extensively about treatment options and should acknowledge the legitimacy of patients' values in deciding about treatment. Ideally, treatment guidelines should include the recommendation that patient values about treatment benefits and side effects should explicitly be elicited at the time a treatment decision needs to be made.

## Figures and Tables

**Table 1:**
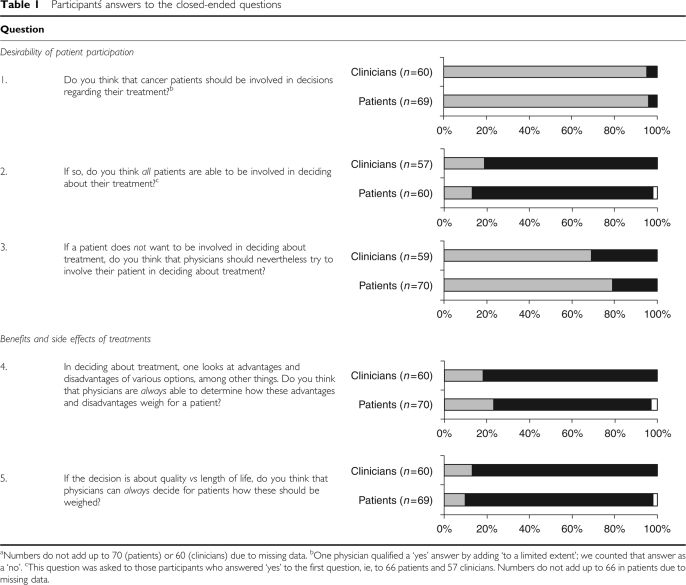
Participantś answers to the closed-ended questions

**Table A1:** 

**Question**	**Heading**	**Subheading**	**Examples of utterances coded**
1. *Do you think that cancer patients should be involved in decisions regarding their treatment?*^a^			
	Conditions for involvement	Information	Being able to understand information about alternatives; right to receive information
		Patient-clinician relationship	Clinician's honesty; patient's trust in clinician; clinician's clarity of information
		Decision	There is a choice to be made
	Roles in decision-making process	Patient	Patient autonomy; patient chooses what he/she considers as quality; patient carries responsibility; patient thinks along
		Clinician	Clinician has the expertise; clinician advises; clinician is responsible
	Outcome of decision-making process		Patient and clinician reach agreement; motivated patient endures the treatment better
	Legal obligation	Informed consent procedure	Need to comply to the informed consent procedure
2. *If so, do you think all patients are able to be involved in deciding about their treatment?*^b^			
	Psychological inability^c^		—
	Emotional barriers		Patient is too anxious; patient is emotionally unstable; patient lacks confidence
	Cognitive barriers		Patient has limited intelligence; patient has difficulties with appraising risks; patient does not understand the information
	Socio-demographic factors		Patient stems from an older generation; patient lacks social support; patient holds particular religious beliefs
	Co-morbidity		Dementia; intellectual disability; psychiatric disorder
	Complex situation		Patient is unfamiliar with decision situation; information is complex
*3. If a patient does not want to be involved in deciding about treatment, do you think that physicians should nevertheless try to involve their patient in deciding about treatment?* d			
	Reason why	Patient: Temporary evasive behaviour	Patient feels panicky; it is important to help prevent regret in patient
		Patient: Responsibility	Patient is (also) responsible; patient has to live with the consequences; patient is autonomous
		Clinician	It is clinician's task; clinician needs patient agreement
		Patient-clinician relationship	Creates mutual trust
	Clinician's devotion to involve patient	To the utmost	Clinician should try to involve patient to the utmost
		Within reason	Clinician should try to involve patient within reason
		Respect patient's wish	Clinician should respect patient's wish not to participate
	Manner in which		Clinician gives information; clinician reassures the patient; clinician takes time; clinician involves significant others of patient; clinician gains patient's trust
*4. In deciding about treatment, one looks at advantages and disadvantages of various options, among other things. Do you think that physicians are always able to determine how these advantages and disadvantages weigh for a patient?* e			
*5. If the decision is about quality vs length of life, do you think that physicians can always decide for patients how these should be weighed?*			
	Yes, the starting point is the…	Patient	Patient trusts the clinician; patient does not understand the situation well enough
		Clinician	Clinician has the expertise; clinician is responsible; clinician has the ability to estimate patient values
		Patient-clinician relationship	Good communication; clinician knows the patient well enough
	No, the starting point is the …	Patient	Individual patients differ too much from one another; patient makes own decision; patient knows best; patientś experience with health or health care differ; patients differ in their acceptance of treatments
		Clinician	Clinician has own subjective stance; clinician cannot infer importance for patient; clinician brings in own emotions
		Patient-clinician relationship	Clinician does not know patient well enough; clinician and patient need to consult together; patient needs to share own values with clinician

a The participants' explanations to this question were only coded in those agreeing to patient involvement.

b The participants' explanations to this question were only coded in those agreeing to patient involvement (question 1) and disagreeing to question 2.

c This category includes references to psychological inabilities in patients that participants did not further specify.

d The participants' explanations to this question were coded both in those answering ‘yes’ and ‘no’ to the question.

e Coding categories for questions 4 and 5 were identical.

## Appendix A Coding scheme

Table A1
